# The Ottawa resident observation form for nurses (O-RON): evaluation of an assessment tool’s psychometric properties in different specialties

**DOI:** 10.1186/s12909-024-05476-1

**Published:** 2024-05-02

**Authors:** Hedva Chiu, Timothy J. Wood, Adam Garber, Samantha Halman, Janelle Rekman, Wade Gofton, Nancy Dudek

**Affiliations:** 1 Department of Medicine, Division of Physical Medicine & Rehabilitation, University of Ottawa, Ottawa, Canada; 2 Department of Innovation in Medical Education, University of Ottawa, Ottawa, Canada; 3 Department of Obstetrics and Gynecology, University of Ottawa, Ottawa, Canada; 4 Department of Medicine, Division of General Internal Medicine, University of Ottawa, Ottawa, Canada; 5 Department of Surgery, Division of General Surgery, University of Ottawa, Ottawa, Canada; 6 Department of Surgery, Division of Orthopedic Surgery, University of Ottawa, Ottawa, Canada; 7 Department of Medicine, Division of Physical Medicine & Rehabilitation), The Ottawa Hospital, University of Ottawa, Ottawa, ON Canada

**Keywords:** Post-graduate medical education, Workplace-based assessment, Inter-professional assessment, Professionalism, Feedback

## Abstract

**Background:**

Workplace-based assessment (WBA) used in post-graduate medical education relies on physician supervisors’ feedback. However, in a training environment where supervisors are unavailable to assess certain aspects of a resident’s performance, nurses are well-positioned to do so. The Ottawa Resident Observation Form for Nurses (O-RON) was developed to capture nurses’ assessment of trainee performance and results have demonstrated strong evidence for validity in Orthopedic Surgery. However, different clinical settings may impact a tool’s performance. This project studied the use of the O-RON in three different specialties at the University of Ottawa.

**Methods:**

O-RON forms were distributed on Internal Medicine, General Surgery, and Obstetrical wards at the University of Ottawa over nine months. Validity evidence related to quantitative data was collected. Exit interviews with nurse managers were performed and content was thematically analyzed.

**Results:**

179 O-RONs were completed on 30 residents. With four forms per resident, the ORON’s reliability was 0.82. Global judgement response and frequency of concerns was correlated (*r* = 0.627, *P* < 0.001).

**Conclusions:**

Consistent with the original study, the findings demonstrated strong evidence for validity. However, the number of forms collected was less than expected. Exit interviews identified factors impacting form completion, which included clinical workloads and interprofessional dynamics.

## Background

As the practice of medicine evolves, medical educators strive to refine the teaching curriculum and find innovative ways to train physicians who can adapt to and thrive within this changing landscape. In 2015, The Royal College of Physicians & Surgeons of Canada published the updated CanMEDS competency framework [1], which emphasizes the importance of intrinsic roles in addition to the skills needed to be a medical expert. These intrinsic roles are important in developing well-rounded physicians, but are less tangible and can be challenging to integrate into traditional assessment formats [2–4]. Knowing this, medical educators are given the task of developing new ways to assess these skills in resident physicians.

Another innovation in medical education is the shift from a traditional time-based curriculum to a competency-based curriculum (or competency-based medical education, “CBME”). This shift allows for an increased focus on a resident’s learning needs and achievements. It encourages a culture of frequent observed formative assessments [5]. This shift calls for assessment tools that accurately reflect a resident’s competence and can be feasibly administered in the training environment.

Workplace-based assessments (WBA) are considered one of the best methods to assess professional competence in the post-graduate medical education curriculum because they can be feasibly administered in the clinical setting [6, 7]. Most WBA relies on physician supervisors making observations of residents. However, restraints of a complex and busy training environment mean that supervisors are not always available to observe some aspects of a resident’s performance. For example, when a resident rounds on patients independently or attends to on-call scenarios in the middle of the night, the physician supervisor may not be present. Physician supervisors may also not be present during multi-disciplinary team meetings where residents participate in the co-management of patients with other health professionals.

On a hospital ward, the health professional that most often interacts with a resident is a nurse. Given this, it makes sense to consider obtaining assessment information from a nurse’s viewpoint. This has the potential to be valuable for several reasons. First, they may provide authentic information about resident performance because residents may perform differently when they know that they are not being directly observed by their physician supervisors [8]. Second, nurses play an integral role in patient care, and often serve as a liaison between patients, their families and physicians regarding daily care needs and changes to clinical conditions. This liaison role provides nurses with a unique perspective on the intrinsic roles of physician competence in patient management, communication, and leadership skills that would also improve collaboration between nurses and physicians [9]. As such, using a WBA tool that incorporates nursing-identified elements of physician competence to assess a resident’s ability to demonstrate those elements in their workplace is important in training future physicians.

Although assessment of resident performance by nurses is captured with multi-source feedback (MSF) tools, there are some concerns if relying solely on this approach, as MSF tools generally present the data as an aggregate score regardless of individual rater roles. This convergence of ratings may not be helpful in feedback settings because it disregards how behaviour can change in different contexts (i.e., the specific situation and the relationship of the rater with the one being rated) [10]. Furthermore, there is evidence that different groups of health professionals rate the same individuals differently, more specifically, there is evidence to suggest that nursing perspectives often differ from other health professionals and physician supervisors [11–16]. When the groups are combined, the perspective of one group can be lost. It is not a weakness that different groups have different perspectives, but it needs to be documented to provide more useful formative feedback. Therefore, there is a need for a tool that uniquely captures the nurses’ perspective of resident performance.

**Fig. 1:**
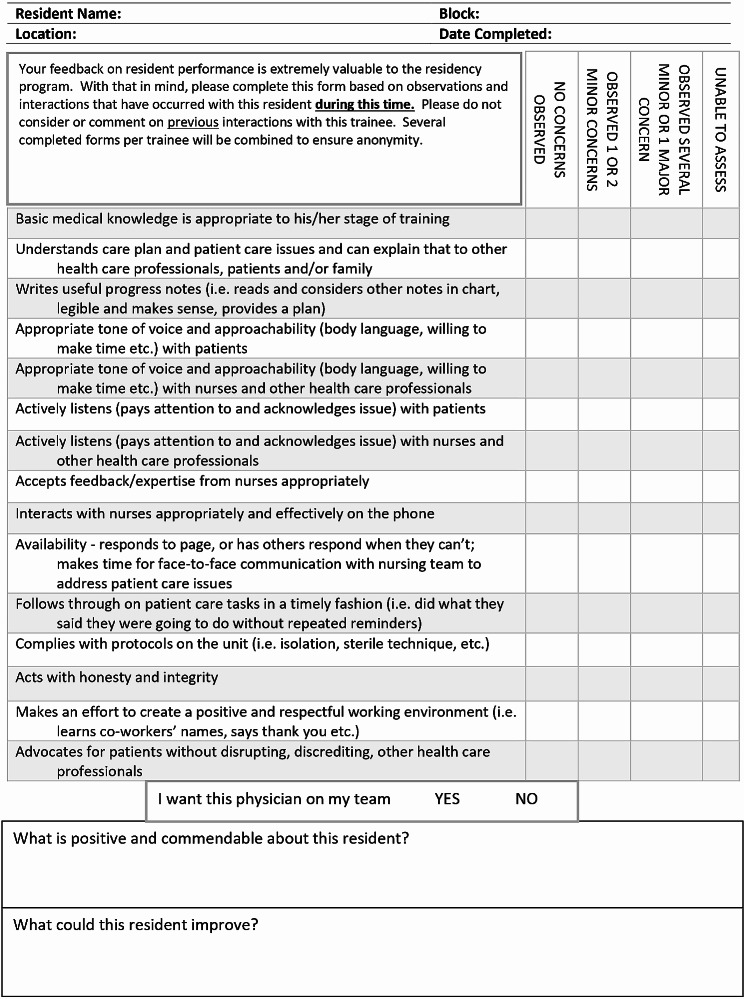
The Ottawa resident observation form for nurses (O-RON)

To address this issue, Dudek et al. (2021) developed The Ottawa Resident Observation Form for Nurses (O-RON), a tool that captures nurses’ assessment of resident performance in a hospital ward environment (Fig. 1). This tool allows nurses to identify concerning behaviours in resident performance. The tool was implemented and studied in the Orthopedic Surgery Residency Program at the University of Ottawa, Canada. Nurses voluntarily completed the O-RON and indicated that it was easy to use. Validity evidence related to internal processes was gathered by calculating the reliability of the scale using a generalizability analysis and decision study. The results showed that with eight forms per resident the reliability of the O-RON was 0.8 and with three forms per resident, the reliability was 0.59. A reliability of 0.8 is considered acceptable for summative assessments [17]. These results suggest that the O-RON could be a promising WBA tool that provides residents and training programs with important feedback on aspects of residents’ performance on a hospital ward through the eyes of the nurses.

The O-RON garnered international interest. Busch et al. translated the O-RON into Spanish and implemented it in two cardiology centres in Buenos Aires [18]. Their findings also demonstrated strong evidence for validity, although they required a higher number of forms (*n* = 60) to achieve high reliability (G coefficient = 0.72).

The demonstrated psychometric characteristics of the tool for these two studies were determined in single specialties. Local assessment culture, clinical setting, interprofessional dynamics and rater experience are some of the factors that can affect how a nurse may complete the O-RON [19–23]. These external factors can lead to measurement errors, which in turn would impact the generalizability and validity of the O-RON. Therefore, further testing is vital to determine whether the O-RON will perform consistently in other environments [24, 25].

The primary objective of this project was to collect additional validity evidence related to the O-RON by implementing it in multiple residency programs including both surgical and medical specialties, which represent different assessment cultures and clinical contexts. However, it became evident throughout the data collection period that the number of completed forms was lower than anticipated. As such, there needed to be shift in focus to also explore challenges surrounding implementation of a new assessment tool in different programs. Therefore, the secondary objective of this study was to better understand the barriers to the implementation of the O-RON.

## Methods

This study sought to assess the psychometric properties of the O-RON in three specialties at the University of Ottawa, Canada, using modern validity theory as a framework to guide the evaluation of the O-RON [25]. The O-RON was used in the Core Internal Medicine, General Surgery, and Obstetrics and Gynecology residency programs at the University of Ottawa. These programs did not have an assessment tool completed exclusively by nurses to evaluate their residents prior to the start of the project. They agreed to provide the research team with the anonymized data from this tool to study its psychometric properties. Ethics approval was granted by the Ottawa Health Science Network Research Ethics Board.

### The Ottawa resident observation form for nurses (O-RON)

Dudek et al. (2021) developed the O-RON through a nominal group technique where nurses identified dimensions of performance that they perceived as reflective of high-quality physician performance on a hospital ward. These were included as items, of which there were 15, on the O-RON. Each item is rated on a 3-point frequency scale (no concerns, minor concerns, major concerns) with a fourth option of “unable to assess”. There is an additional “yes/no” question regarding whether the nurse would want to work with the resident as a team member (“global assessment question”) and a space for comments.

### Procedure

Residents from the three residency programs were provided a briefing by their program director on the use of the O-RON prior to the start of the project. Nurses on the internal medicine, general surgery, and obstetrics wards at two hospital campuses were asked to complete the O-RON for the residents on rotation. Nurse managers reviewed the form with the nurses at the start of the project and were available for questions. This was consistent with how the tool was used in the original study. At the end of each four-week rotation, 10 O-RON forms per resident were distributed to the nurse manager, who then distributed them to their nurses. Nurses were assigned a code by the nurse manager so that they could anonymously complete the forms. Any nurse who felt that they would like to provide an assessment on a resident, received a form to complete and returned it to the nurse manager within two weeks. The completed forms were collected by the research assistant at the two-week mark who collated the data for each resident and provided a summary sheet to their program director. The research assistant assigned a code for each resident and recorded the anonymized O-RON data for the study analysis.

### Sample size

In the original study [26] of the O-RON the results demonstrated a strong reliability coefficient (0.80) with a sample of eight forms per resident. Using the procedure described by Streiner and Norman [24], an estimate of 256 forms in total was needed to achieve a desired reliability of 0.80 with a 95% confidence interval of +/- 10%. Typically, there were 16 residents ranging from PGY1-3 participating in a general internal medicine ward, 16 residents ranging from PGY 1–5 participating in a general surgery ward, and eight residents ranging from PGY1-5 participating in a labour and delivery ward at any time. To have at least 256 forms per specialty and considering that nurses were unlikely to complete 10 forms on each resident each time and fluctuations in resident numbers between rotations is expected, a collection period of six months was established.

### Response to low participation rate

The completion rate was closely monitored throughout the collection period. There was a low rate of participation after six rounds of collection. In response, we initiated improvement processes including (a) displaying photos of the residents with their names in the nursing office, (b) displaying a poster about the project as a reminder for the nurses in the nursing office, (c) reaching out to nurse managers to review the project. We also extended the collection period for additional three rotations for a total of nine rotations to allow time for the improvement processes to work.

At the end of the extended collection period, we conducted semi-structured interviews with each nurse manager individually at each of the O-RON collection sites to further explore reasons behind low participation rate.

### Quantitative analyses

Analyses were conducted using SPSS v27 statistical software. Rating response frequencies were calculated across scale items and “yes/no” frequencies were calculated for the global assessment question. Chi-square tests were conducted on each item against the global assessment response to determine the effect of concerns on the global assessment. Total O-RON score was calculated for the purposes of data analysis by counting the number of items that had a minor or major rating and dividing by the number of items that had a valid rating. A higher score indicated more concerns. Invalid rating items with either “unable to assess” as a response or left blank were excluded from this analysis. Tests of between-subjects effects were conducted between total O-RON score and the global assessment rating.

The reliability of the O-RON was calculated using a generalizability analysis (g-study) and the number of forms required for an acceptable level of reliability was determined through a decision study. These outcomes contributed to validity evidence related to internal processes.

A g-study calculates variance components, which can be used to derive the reliability of the O-RON. Variance components are associated with each facet used in the analysis and reflect the degree to which overall variance in scores is attributed to each facet. For this study, this was calculated using the mean total scores, which were analyzed using a between subjects ANOVA with round as a grouping facet, and people and forms as nested facets. Using the results from the generalizability analysis, a decision study derives estimates of reliability based on varying the facets used in the analysis. For our study, we varied the number of forms per resident to understand its impact on the reliability of the O-RON.

### Qualitative analyses

Semi-structured exit interviews were conducted by the study principal investigator (HC) with each nurse manager. They were voice-recorded and transcribed into text documents. Using conventional content analysis, interview content was thematically analysed and coded by two of the study’s co-investigators (HC and ND) independently. The codes were compared between the two researchers and a consensus was met. This coding structure was then used to code all six interviews.

## Results

### Quantitative

180 O-RONs were completed on 30 residents over the study period with an average of six forms per resident (range = 1–34). The large range is due to some residents being assessed on more than one rotation. One form was excluded from analysis because it had a value of “could not assess” for every item. A total of 179 O-RONs were included for analysis.

The Obstetrics units had the highest frequency of O-RONs completed (74.3%), followed by General Surgery (16.2%), and Internal Medicine (9.5%). Due to the small numbers within each specialty, subsequent analysis was done on the aggregate data.

Across forms and items, the frequency of reported rating in descending order was “no concerns” (80.7%), “minor concerns” (11.5%), “unable to assess” (3.0%), and “major concerns” (1.9%). Blank items accounted for 2.9% of responses. For the global assessment rating, 92.3% of valid responses were “yes” for whether they wanted this physician on their team (Table 1).

**Table 1:** Rating response frequency across forms (items 1–15 and global rating)

Scale Items	Response Rate				
	No concerns (0)	Minor Concerns [1]	Major Concerns [2]	Unable to Assess	No Response
1. Basic medical knowledge is appropriate to his/her stage of training	146 (81.6%)	18 (10.1%)	8 (4.5%)	3 (1.7%)	4 (2.2%)
2. Understands care plan and patient care issues and can explain that to other health care professionals, patients and/or family	149 (83.2%)	19 (10.6%)	3 (1.7%)	2 (1.1%)	6 (3.4%)
3. Writes useful progress notes (i.e. reads and considers other notes in chart, legible and makes sense, provides a plan)	151 (84.4%)	6 (3.4%)	2 (1.1%)	12 (6.7%)	8 (4.5%)
4. Appropriate tone of voice and approachability (body language, willing to make time etc.) with patients	141 (78.8%)	27 (15.1%)	5 (2.8%)	0	6 (3.4%)
5. Appropriate tone of voice and approachability (body language, willing to make time etc.) with nurses and other health care professionals	140 (78.2%)	32 (17.9%)	4 (2.2%)	0	3 (1.7%)
6. Actively listens (pays attention to and acknowledges issue) with patients	143 (79.9%)	22 (12.3%)	4 (2.2%)	4 (2.2%)	6 (3.4%)
7. Actively listens (pays attention to and acknowledges issue) with nurses and other health care professionals	133 (74.3%)	34 (19.0%)	4 (2.2%)	1 (0.6%)	7 (3.9%)
8. Accepts feedback/expertise from nurses appropriately	130 (72.6%)	35 (19.6%)	4 (2.2%)	4 (2.2%)	6 (3.4%)
9. Interacts with nurses appropriately and effectively on the phone	141 (78.8%)	20 (11.2%)	3 (1.7%)	11 (6.1%)	4 (2.2%)
10. Availability - responds to page, or has others respond when they can’t; makes time for face-to-face communication with nursing team to address patient care issues	142 (79.3%)	19 (10.6%)	5 (2.8%)	9 (5.0%)	4 (2.2%)
11. Follows through on patient care tasks in a timely fashion (i.e. did what they said they were going to do without repeated reminders)	137 (76.5%)	28 (15.6%)	4 (2.2%)	5 (2.8%)	5 (2.8%)
12. Complies with protocols on the unit (i.e. isolation, sterile technique, etc.)	154 (86.0%)	10 (5.6%)	0	8 (4.5%)	7 (3.9%)
13. Acts with honesty and integrity	162 (90.5%)	5 (2.8%)	0	6 (3.4%)	6 (3.4%)
14. Makes an effort to create a positive and respectful working environment (i.e. learns co-workers’ names, says thank you etc.)	153 (85.5%)	20 (11.2%)	3 (1.7%)	2 (1.1%)	1 (0.6%)
15. Advocates for patients without disrupting, discrediting, other health care professionals	144 (80.4%)	14 (7.8%)	1 (0.6%)	14 (7.8%)	6 (3.4%)
	Yes		No		No Response
Global judgement question	143 (92.3%)		12 (7.7%)		24

Note. Summed across total 179 forms (contains multiple raters and observations per resident)

In terms of item-level analysis, nurses reported the least concern for item 13 (“acts with honesty and integrity”) (90.5% - no concerns). They reported the most major concerns for item 1 (“basic medical knowledge is appropriate to his/her stage of training”) (4.5% - major concerns), and the most overall concerns for item 8 (“Accepts feedback/expertise from nurses appropriately”) (21.8% - minor + major concerns). The raters were most frequently unable to assess item 15 (“advocates for patients without disrupting, discrediting, other HCP”) at 7.8%.

2 × 2 comparison tests were used to assess the presence of concern as a function of their response to the global assessment question (Table 2). Since there was only a small number of major concerns for each item, minor and major concerns were combined (“any concerns”). All items except four (items 10, 12, 13 and 14) showed a statistically significant difference (*P* < 0.01). Tests of between-subjects effects was used to compare between total O-RON score and response to the global assessment question, which showed a correlation between global response and frequency of concerns (*r* = 0.627, *P* < 0.001).

**Table 2:** Frequency of concerns to “I want this physician on my team” for items

	On Team	No Concerns	Any Concerns	Total	Fisher’s Exact Test
Item 1	No	2	10	12	< 0.001
	Yes	132	5	137	
	Total	134	15	149	
Item 2	No	3	9	12	< 0.001
	Yes	134	4	138	
	Total	137	13	150	
Item 3	No	5	5	10	< 0.001
	Yes	131	1	132	
	Total	136	6	142	
Item 4	No	3	8	11	< 0.001
	Yes	124	16	140	
	Total	127	23	151	
Item 5	No	2	9	11	< 0.001
	Yes	125	17	142	
	Total	127	26	153	
Item 6	No	3	8	11	< 0.001
	Yes	132	7	139	
	Total	135	15	150	
Item 7	No	1	11	12	< 0.001
	Yes	124	13	137	
	Total	125	24	149	
Item 8	No	4	8	12	< 0.001
	Yes	121	15	136	
	Total	125	34	148	
Item 9	No	5	7	12	< 0.001
	Yes	125	9	134	
	Total	130	16	146	
Item 10	No	8	4	12	0.017
	Yes	124	10	134	
	Total	132	14	146	
Item 11	No	4	7	11	< 0.001
	Yes	123	16	139	
	Total	127	23	150	
Item 12	No	7	2	9	0.045
	Yes	132	4	136	
	Total	139	6	145	
Item 13	No	9	1	10	0.068
	Yes	128	0	138	
	Total	147	1	148	
Item 14	No	8	3	11	0.107
	Yes	128	14	142	
	Total	136	17	153	
Item 15	No	1	7	8	< 0.001
	Yes	133	2	135	
	Total	134	9	143	

The g-study results showed that people (object of measurement) accounted for 54% of the variance. Rotation did not account for any variance indicating that ratings were similar across all nine rotations. The decision study results showed that with three forms per resident, the reliability was 0.78 and with four forms, the reliability was 0.82.

### Qualitative

#### Factors impacting the implementation of the O-RON

Five themes were identified as factors that had an impact, whether positive or negative, on the implementation of the O-RON (Table 3).

**Table 3:** Factors impacting the implementation of the O-RON and suggestions for improvement

Factors impacting O-RON implementation	Suggestions for improvement
Strong project lead on the unit	Mixed leadership roles
Familiarity with residents	Increase familiarity between nurses and residents (i.e. more in-person rounds, involving the residents in the distribution of O-RONs)
Nursing workload	Transparent feedback procedure
Work experience of nurses	Format of the O-RON
Culture of assessment	

#### Strong project lead on the unit

Units where clinical managers described strong involvement of a lead person (usually themselves) who was persistent in reminding nurses to complete O-RONs and were passionate about using the tool had higher completion O-RON rates. Conversely, if there was not such a strong lead, there was a much lower O-RON completion rate.

*“If I was to step away from this position and it was a different manager coming in, would they do the same that I would do in this process, I don’t know. So[…]I know it works okay for me because […] I don’t see it as a huge investment of time[…]but if I’m off or I’m not here[…]it’s finding a nurse who would be responsible to do it.” (Participant 2)*.

*“[…]from the leadership perspective, we talk about it, but we don’t own it […] The feedback doesn’t change anything to me as a leader, as a manager. […] Not that I don’t concentrate on the O-RON, I do talk about it, but I’m not passionate about it.” (Participant 4)*.

#### Familiarity with residents

Clinical managers expressed the importance of having collegial relationships with the residents. This was usually facilitated by having a smaller number of residents or having in-person ward rounds. Because of this, the nurses knew the residents better, had more time to work with them personally, and were able to match their faces to their names more frequently. Conversely, if a unit employed virtual rounds, had a lot of residents, or mainly used technology to communicate with residents, the nurses were unfamiliar with the residents and felt they were not able to comment as easily on resident performance.

*“So with our group, […] our […] residents, is tiny. There’s two of them on at a time in a month. Maybe only one. So, […] they’re here 24 hours, with our nurses, working, they get to know each other quite well, so, that could be a contributing factor potentially.” (Participant 2)*.

*“Where before we used to have rounds and the residents would come and the staff would come, so we could have that connection with the resident. We could put a face to them, a name to them. We knew who they were. Where, with EPIC* [electronic medical record system], *first of all the nurses don’t attend EPIC rounds. We don’t see the residents, we don’t see the staff. Like I have no idea, who […] is because I don’t see him. So, it’s very difficult for me to do an evaluation on someone I have not met, not seen, and only see through EPIC. A lot of the conversations the nurses have are also through EPIC, they’ll send an EPIC chat. The resident will email back. So, you know, it’s missing that piece.” (Participant 1)*.

#### Nursing workload

Clinical managers mentioned that completing the O-RON was an additional item to their existing full workload. This was largely driven by an overall shortage of staff and a large number of new nurses joining the units. The new nurses are trying to learn new protocols and clinical skills and had little capacity to do extra work.

*“I mean every day we are working short, right? We’re missing one or two nurses. I have nurses from other units, I have nurses that have never been here. So yes, I could see how that would have contributed to having a lower response.” (Participant 1)*.

*“I’m going to say about 60% of our staff have less than one year experience and we’ve also re-introduced RPNs to the unit. And so the unit right now is really burdened with new staff. But it’s not only new staff, but it’s new staff whose skillset are not as advanced as what they potentially would have been five years ago. And so the staff are really concentrating on beefing up their skillset, just really integrating into the unit. And so, there is really not a lot of thought or concentration necessarily on trying to do the extras, such as doing the surveys.” (Participant 4)*.

#### Work experience of nurses

In addition to new nursing staff having less time for non-essential tasks, clinical managers also pointed out that newer nurses tended to be more hesitant to comment on a resident’s performance compared to a more experienced nurse.

*“A lot of junior staff that I don’t know if they would take that initiative to […] put some feedback on a piece of paper for a resident even though it’s almost untraceable to them. You know, a little bit more timid and shy.” (Participant 6)*.

*“Most of them [those who filled out the form] were the […]mid-career nurses. So, right now, my mid-career nurses have been around for five to ten years. […] And so those nurses are the ones who are still very engaged, wanting to do different projects. Those were the nurses that were doing it, it was not the newer hires, and it was not the nurses who have been here for, you know, 20 + years.” (Participant 4)*.

#### Culture of assessment

All clinical managers interviewed noted that there was not a strong culture of nurses providing any feedback or assessment of residents prior to the implementation of the O-RON. There may have been informal discussions and feedback, but there was no formal process or tool.

#### Suggestions for improvement

Four suggested areas for improvement of the implementation of the O-RON were identified (Table 3).

#### Mixed leadership roles

Clinical managers suggested that having physicians promote the O-RON in addition to themselves may be helpful.

*“But I’m even thinking, like if it didn’t just come from me, if the staff* [doctor] *would come around and say, “Hey guys, I would really appreciate it.” […] say if it came just from me, from oh the manager is asking for us to fill out another sheet, or something to that effect. It may help a little bit.” (Participant 1)*.

*“I think at the huddle, if one of you can come (Staff physician), although we mention it, but I think it would be important, even if it’s only once a month, you know. […] Or you know, come on the unit anytime and just you know, remind the nurses.” (Participant 3)*.

#### Increase familiarity between nurses and residents

Clinical managers suggested increasing familiarity between nurses and residents by having more in-person rounds where residents regularly attend and involving the residents in the distribution of O-RONs.

*“My recommendation would be to bring back rounds, in-person rounds. Also, it would be nice if we would have like an introduction. ‘This is the resident for Team C,’ you know something to that effect. I know they come around and they sit, and they look at EPIC and they chat, but we sometimes don’t make the connection of who is this resident, you know, what team is he part of.” (Participant 1)*.

*“I guess maybe a suggestion would be to have the residents go around, and not every single day, but maybe once a week, prioritise 30 minutes and take their own surveys and go up to the nursing staff and say, “Hey, I’m looking for your feedback, will you complete this survey for me?” And then hand the nurse the survey that relates directly to that particular resident.” (Participant 4)*.

#### Transparent feedback procedure

Clinical managers highlighted the importance of having a clear loop back procedure that allows the nurses to know that their feedback is being reviewed and shared with the residents. They felt that this is very important for maintaining nursing participation in resident assessment.

*“I guess the one question is, they fill this in, but now we’re getting to a point of, how do we know that information or how is that information getting to the residents? What sort of structure is that? So that at least I can have a conversation explaining that yeah, when you fill this in, this is the next steps that happen of how it loops back with the individuals. So I think the further along we get into this and not having that closed loop on it, we may start to lose some engagement because then their maybe not going to see a worth or value to doing it.” (Participant 2)*.

#### Format of the O-RON

Some clinical managers felt having different formats of the O-RON available for use (paper and digital) may increase engagement. They pointed out that some nurses really like the option of a digital version of surveys that they have used in different projects. On the other hand, others pointed out that some of their staff preferred a paper form.

## Discussion

WBAs that rely on observations by physician supervisions is a predominant method used to assess professional competency in the post-graduate medical education curriculum [7]. However, in a complex training environment where supervisors are unavailable to observe certain aspects of a trainee’s performance, nurses are well-positioned to do so. The O-RON was developed to capture nurses feedback, which is critical in identifying and fostering the development of physician characteristics that improve collaboration between nurses and physicians [9]. Our study assessed the use of the O-RON in three different residency programs at the University of Ottawa to gather more validity evidence and allow us to generalize results to multiple contexts.

As in the original study, our findings demonstrated strong validity evidence for internal processes, which was demonstrated by the calculation of reliability using the generalizability analysis and decision study. With only four forms per resident, the O-RON had a reliability of 0.82, and with three forms, the O-RON had a reliability of 0.78. A reliability range of 0.8–0.89 is considered acceptable for moderate stakes summative assessments and a reliability range of 0.7–0.79 is considered acceptable for formative assessments [17]. The results of the 2 × 2 comparison tests highlighted the correlation between global assessment and presence of concern, which reflected that nurses would more likely want to work with a physician who showed no concerning behaviour on the O-RON items. This further supports the consistency of the tool in identifying concerning behaviour through the eyes of nurses.

However, in our study we had substantially fewer forms completed than in the original study (180 forms for 30 residents over nine months versus 1079 forms for 38 residents over 11 months) and less than the intended sample size of 256 forms per specialty. Because of that, we were only able to analyze the data as an aggregate rather than per specialty and were not able to make comparisons between specialty groups. Nonetheless, there was a sufficient number of submitted forms to perform the generalizability analysis and the dependability analysis allowed us to estimate the reliability of the O-RON with a range of submitted forms. Furthermore, the resulting reliability was greater than was obtained in the original study [26].

To better understand the reasons behind this difference, we conducted semi-structured interviews with the clinical managers on each unit individually. Five major themes were identified that had an impact on the implementation of the O-RON. Better implementation occurred when there was strong leadership for the implementation of the tool, there were a higher number of experienced nurses, and the nurses knew the residents. When these factors were absent, uptake of the tool was limited. Additionally, heavy clinical workloads related to staffing shortages caused both by the COVID pandemic and the current nursing staffing crisis in Canada had a significant negative impact. Furthermore, certain COVID protocols and the implementation of the electronic health record made a lot of nurse-resident interaction more virtual instead of in-person. It is also worth noting that the wards in the original study had an established culture of feedback collected by the clinical managers who reported it on a regular basis using their own form to the residency program director. This may also have contributed to the more successful implantation of the O-RON in the original study.

The barriers to implementation we identified in our study are consistent with the literature on challenges facing implementation of new assessment tools. Local assessment culture, clinical setting, interprofessional dynamics, leadership engagement and time constraint issues have all been previously identified [27–29]. Our study was able to additionally highlight nursing suggestions to address these barriers, which include mixed leadership roles, ways to improve collegial familiarity, and feedback transparency (Table 3).

Despite the challenges identified, clinical managers were appreciative of the O-RON as an avenue for nurses to be assessors and felt that it was a valuable tool. That, in combination with its growing evidence for validity, suggest that future work should be targeted towards addressing the barriers prior to implementation of the O-RON. Our study participants offered several suggestions for this. They also emphasized the importance on ensuring that nurses are made aware of how their assessments will be provided and followed up on with residents.

Our study has limitations. First, there was a relatively smaller number of completed O-RONs compared to what we had anticipated. Because of that, we needed to aggregate the data between all specialties for further analysis rather than analyse them separately. This also led us to pursue the qualitative portion of our study, which characterized why this was the case. This new information may be beneficial for future work. Second, this study was performed in a single university and three specific specialties. To generate further evidence for validity of the O-RON as an assessment tool, implementing the O-RON at different institutions and specialties should be considered.

## Conclusions

The O-RON is a useful tool to capture nurses’ assessment of resident performance. The findings of our study demonstrated reliable results in various clinical settings thus adding to the validity of the results. However, understanding the assessment environment and ensuring it has the capacity to perform this assessment is crucial for its successful implementation. Future research should focus on how we can create conditions whereby implementing this tool is feasible from the perspective of nurses.

## Data Availability

The datasets used and/or analysed during the study are available from the corresponding author on reasonable request.

## Abbreviations

CBME: Competency-based medical education
O-RON: Ottawa Resident Observation Form for Nurses
WBA: Workplace-based assessment
